# Incidence and Effects of Acquisition of the Phage-Encoded *ssa* Superantigen Gene in Invasive Group A *Streptococcus*

**DOI:** 10.3389/fmicb.2021.685343

**Published:** 2021-06-04

**Authors:** Chuan Chiang-Ni, Yen-Shan Liu, Chieh-Yu Lin, Chih-Yun Hsu, Yong-An Shi, Yi-Ywan M. Chen, Chih-Ho Lai, Cheng-Hsun Chiu

**Affiliations:** ^1^Department of Microbiology and Immunology, College of Medicine, Chang Gung University, Taoyuan, Taiwan; ^2^Graduate Institute of Biomedical Sciences, College of Medicine, Chang Gung University, Taoyuan, Taiwan; ^3^Molecular Infectious Disease Research Center, Chang Gung Memorial Hospital, Linkou, Taiwan; ^4^Department of Orthopedic Surgery, Chang Gung Memorial Hospital, Linkou, Taiwan; ^5^Division of Pediatric Infectious Diseases, Department of Pediatrics, Chang Gung Memorial Hospital, Linkou, Taiwan

**Keywords:** group A *Streptococcus*, streptococcal superantigen, SSA, CovR/CovS, superantigen, invasive GAS infection

## Abstract

The acquisition of the phage-encoded superantigen *ssa* by scarlet fever-associated group A *Streptococcus* (*Streptococcus pyogenes*, GAS) is found in North Asia. Nonetheless, the impact of acquiring *ssa* by GAS in invasive infections is unclear. This study initially analyzed the prevalence of *ssa*+ GAS among isolates from sterile tissues and blood. Among 220 isolates in northern Taiwan, the prevalence of *ssa*+ isolates increased from 1.5% in 2008–2010 to 40% in 2017–2019. Spontaneous mutations in *covR*/*covS*, which result in the functional loss of capacity to phosphorylate CovR, are frequently recovered from GAS invasive infection cases. Consistent with this, Phostag western blot results indicated that among the invasive infection isolates studied, 10% of the *ssa*+ isolates lacked detectable phosphorylated CovR. Transcription of *ssa* is upregulated in the *covS* mutant. Furthermore, in *emm*1 and *emm*12 *covS* mutants, *ssa* deletion significantly reduced their capacity to grow in human whole blood. Finally, this study showed that the *ssa* gene could be transferred from *emm*12-type isolates to the *emm*1-type wild-type strain and *covS* mutants through phage infection and lysogenic conversion. As the prevalence of *ssa*+ isolates increased significantly, the role of streptococcal superantigen in GAS pathogenesis, particularly in invasive *covR/covS* mutants, should be further analyzed.

## Introduction

*Streptococcus pyogenes* [group A *Streptococcus* (GAS)] is a gram-positive bacterium that causes diseases like pharyngitis, pyoderma, scarlet fever, necrotizing fasciitis, and toxic shock syndrome. In 2011, a scarlet fever outbreak was reported in Hong Kong (Hsieh and Huang, 2011; Tse et al., 2012). The scarlet fever isolates typically harbor phage-associated superantigen genes *ssa* and *speC*, and the DNase gene *spd1* (Tse et al., 2012; Ben Zakour et al., 2015). Luk et al. (2012) found insufficient evidence to support the association between increased scarlet fever incidence or severity and a particular *emm* type, virulence gene profile, or the presence of specific foreign genetic elements. Nonetheless, Davies et al. (2015) showed that *ssa* is absent in the clade not associated with scarlet fever, but variably present (95% isolates) in scarlet fever-associated clades, suggesting that *ssa* acquisition could be potentially related to the expansion of scarlet fever-associated *emm*12-type clones within the Hong Kong population.

An recent increased incidence of scarlet fever has also been reported in South Korea, Singapore, England, and Germany (Turner et al., 2016; Park et al., 2017; Brockmann et al., 2018; Kim and Cheong, 2018; Lamagni et al., 2018; Walker and Brouwer, 2018; You et al., 2018; Yung and Thoon, 2018). Park et al. (2017) showed that *emm*4, *emm*28, *emm*1, and *emm*3 contributed to scarlet fever in South Korea. Notably, antibiotic resistance was uncommon in these scarlet fever-associated isolates (Park et al., 2017). Interestingly in England, the most prevalent isolates recovered during a national increase in scarlet fever incidence in 2016 were an emergent clone of the *emm*1 M1T1 genetic lineage, designated M1T1_UK_, which was characterized by increased production of the phage-encoded SpeA superantigen (Lynskey et al., 2019). This contrasts with the results in Hong Kong and Beijing where during an increased incidence of scarlet fever cases in 2011. The most prevalent isolates recovered were polyclonal *emm*12 strains and the vast majority of which were characterized by the presence of the phage-encoded streptococcal superantigen (SSA; Davies et al., 2015). These findings show that *emm* type, antibiotic-resistant phenotype, and superantigen gene in scarlet fever-associated isolates vary from country to country; therefore, the specific factors leading to scarlet fever resurgence are not completely understood.

Streptococcal superantigen is a 260-residue protein with 60% sequence identify to that of staphylococcal enterotoxin B (SEB; Reda et al., 1994). Brouwer et al. (2020) showed that glutathione from streptolysin O (SLO)-lysed host cells not only enhances SSA production but also activates its superantigen activity. *ssa* has been detected in the toxic shock syndrome related M3 isolates (Mollick et al., 1993), suggesting it to be a potential virulence factor of GAS. As the prevalence of *ssa*+ GAS isolates was >70% in certain geographic areas (Li et al., 2020a), the effects of *ssa* acquisition or *ssa*+ isolate dissemination on GAS diseases need to be further clarified.

Previous studies have reported that isolates with spontaneous mutations in the *covR*/*covS* operon are highly related to severe diseases such as necrotizing fasciitis and toxic shock syndrome (Ikebe et al., 2010; Friaes et al., 2015). CovR/CovS is a two-component regulatory system in GAS, and intracellular CovR is phosphorylated by the sensor kinase CovS (Trevino et al., 2009; Tran-Winkler et al., 2011). Mutations in the *covR*/*covS* operon increase the production of virulence factors such as hyaluronic acid capsule, M protein, SLO, and streptokinase (Tran-Winkler et al., 2011; Chiang-Ni et al., 2017; Horstmann et al., 2018). Furthermore, CovR/CovS also regulates the expression of phage-related genes such as DNase *sda1* (Walker et al., 2007). Increased Sda1 expression by a *covS* mutant promoted bacterial escape from immune clearance by degrading the DNA structure of the neutrophil extracellular traps (Walker et al., 2007).

In this study, we found that four *ssa*+ isolates lacked phosphorylated CovR, and *ssa* transcription was upregulated in these isolates. In line with the repression in *ssa* and *slo* transcription by the CovR/CovS system, *ssa* deletion in the *emm*1 and *emm*12 *covS* mutants attenuated bacterial growth activity in human blood, suggesting that *ssa* acquisition by *covS* mutants or spontaneous mutations in the *covR/covS* operon of *ssa*+ isolates could enhance bacterial survival during infection. As the prevalence of *ssa*+ isolates dramatically increased in northern Taiwan, the impact of the dissemination of *ssa*+ isolates on our society should be continuously monitored and studied.

## Materials and Methods

### Bacterial Isolates and Culture Conditions

Group A *Streptococcus* isolates from sterile tissues [ascites (2), blood (199), deep tissue (14), pleural fluid (3), and synovial fluid (2)] collected during 2008–2019 (220 isolates) at the LinKou Chang Gung Memorial Hospital (Taiwan) were included in this study. The *emm*1 wild-type A20 strain, its *covR* mutant, *covS* mutant AP3, and the CovS kinase-inactivated (CovS_H280A_) mutant were described previously (Chiang-Ni et al., 2016, 2017). GAS strains were cultured on trypticase soy agar with 5% sheep blood or in tryptic soy broth (Becton, Dickinson and Company; Sparks, MD, United States) supplemented with 0.5% yeast extract (TSBY). *Escherichia coli* DH5α was purchased from Yeastern Biotech Co., LTD. (Taipei, Taiwan) and was cultured in Luria-Bertani (LB; Becton, Dickinson and Company; Sparks, MD, United States) broth at 37°C with vigorous aeration. When appropriate, chloramphenicol (25 μg/ml and 3 μg/ml for *E. coli* and GAS, respectively) and spectinomycin (100 μg/ml) were used for selection. This study was approved by the Institutional Review Board (201900274B0 and 202000479B0) of Chang Gung Memorial Hospital, Taiwan.

### Erythromycin and Clindamycin Susceptibility Test

The susceptibility of GAS isolates to erythromycin and clindamycin was determined using a disk diffusion assay according to the Clinical and Laboratory Standards Institute Guideline (Clinical and Laboratory Standards Institute, 2018). Inducible clindamycin resistance in GAS isolates was determined using the D test with erythromycin and clindamycin disks. A flattened inhibition zone around the clindamycin disk proximal to the erythromycin disk was considered a positive result.

### DNA Manipulations

Genomic DNA was extracted using a previously described method (Chiang-Ni et al., 2019a), and *emm* typing, PCR amplification, and DNA sequencing were performed according to the protocol from the Centers for Disease Control and Prevention.^1^ The *ssa* and erythromycin resistance genes (*mefA*, *ermB*, and *ermTR*) were screened for by PCR amplification using previously described primers (Table 1; Meisal et al., 2010; Villasenor-Sierra et al., 2012).

**Table 1:** Primers used in this study.

Primer	Use	Sequence (5'-3')^a^	Reference or source
ermB-1	PCR	cgagtgaaaaagtactcaacc	Villasenor-Sierra et al., 2012
ermB-4		agtaacggtacttaaattgtttac	
ermTR-1	PCR	atagaaattgggtcaggaaaagg	Villasenor-Sierra et al., 2012
ermTR-4		ccctgtttacccatttataaacg	
mefA-1	PCR	agtatcattaatcactagtgc	Villasenor-Sierra et al., 2012
mefA-2		ttcttctggtactaaaagtgg	
ssa-BamHI-F	Construction	gcgggatccgtgagcaaatggccaagaat	This study
ssa-BamHI-R		gcgggatccgtaagcggcagaatcgaaat	
ssa-SacII-F	Construction	tccccgcggtaaaagaaaataactttatg	This study
ssa-SacII-R		tccccgcggcatttggctacctcttatat	
vec78_cat-F-sacII	Construction/Southern blot	tccccgcgggatagatttatgatatag	Chiang-Ni et al., 2018
vec78_cat-R-sacII		tccccgcggatttattcagcaagtctt	
ssa (qPCR)-F	qPCR	cctactccagaacaattaaaca	This study
ssa (qPCR)-R		ggatcttacattagtcccttctac	
gyrA-F-3	qPCR	cgtcgtttgactggtttgg	Chiang-Ni et al., 2016
gyrA-R-3		ggcgtgggttagcgtattta	

a Underline: restriction enzyme site.

### Southern Blot Hybridization

Group A *Streptococcus* chromosomal DNA was digested using *Hin*dIII, and the DNA fragments were resolved on 0.9% agarose gel. The PCR product of the chloramphenicol cassette (716 bp; Table 1) was labeled with alkaline phosphatase as the probe, and DNA hybridization was performed according to the manufacturer’s instructions (AlkPhos Direct Labeling and Detection System; GE Healthcare UK Limited; Amersham, United Kingdom). The signal was detected using a Gel Doc XR+ system (Bio-Rad; Hercules, CA, United States).

### *ssa* and *covS* Isogenic Mutant Construction

*ssa* and flanking upstream and downstream regions (1805 bp) were amplified using primers ssa-*Bam*HI-F and ssa-*Bam*HI-R (Table 1), and the PCR product was ligated into a T-A cloning vector (Yeastern Biotech). *ssa* was removed by PCR with reverse primers ssa-*Sac*II-F and ssa-*Sac*II-R (Table 1) and replaced by the chloramphenicol cassette from Vector78 (Tsou et al., 2010) at the *Sac*II site. The *ssa* knockout DNA fragment was sub-cloned into the temperature-sensitive vector pCN143 (Chiang-Ni et al., 2016) at the *Bam*HI site (designed as pCN211). The plasmids pCN211 and pCN160 (Chiang-Ni et al., 2019a) were transformed into *emm*12 SPY128 and *emm*1 SPY131, respectively, by electroporation for allelic exchange, and the transformants were selected using 3 μg/ml chloramphenicol as described previously (Chiang-Ni et al., 2019b). *ssa* and *covS* deletion in the selected transformants was confirmed by Sanger sequencing. To construct *covS* and *ssa* double mutants, pCN160 was transformed into *ssa* mutants. *covS* deletion in these *ssa* mutants was selected according to the encapsulated phenotype and confirmed by Sanger sequencing.

### RNA Manipulation and Quantitative PCR Analysis

RNA extraction and reverse transcription were performed as previously described (Chiang-Ni et al., 2009). The bacterial strains were cultured for 6 h (exponential phase) and 8 h (stationary phase), and total RNA was extracted for analysis. Quantitative PCR was performed in a 20 μl mixture containing 1 μl cDNA, 0.8 μl primer (10 μM), and 10 μl Sensifast Lo-ROX premixture (Bioline, Ltd.; London, United Kingdom) according to the manufacturer’s instructions. Three biological replicates were performed, and *ssa* expression level was normalized to that of *gyrA* and analyzed using the threshold cycle (∆∆*C_T_*) method (Roche LightCycler® 96 System; Roche Molecular Systems, Inc.; Pleasanton, CA, United States). All values from the control and experimental groups were divided by the mean value of the control samples before statistical analysis (Valcu and Valcu, 2011). Primers used to detect *ssa* (Table 1) and *gyrA* were designed using Primer3 (v.0.4.0) according to the HKU360 sequence (NCBI accession no. CP003901.1).

### Phostag Western Blot Hybridization and Western Blot Analysis

Phostag western blotting was performed as previously described (Chiang-Ni et al., 2019a). Briefly, 10 μg bacterial total protein was mixed with 6× loading dye (without boiling) and separated using 10% SDS-PAGE containing 10 μM Phostag (Wako Pure Chemical Industries, Ltd.; Richmond, VA, United States) and 0.5 M MnCl_2_ for 120–140 min at 100 V at 4°C. For detecting SLO, 30 μl bacterial culture supernatants were mixed with 6× protein loading dye and separated by 12% SDS-PAGE. The separated proteins were transferred onto a polyvinylidene difluoride (PVDF) membrane (Millipore; Billerica, CA, United States), which was blocked with 5% skim milk PBST buffer (PBS containing 0.2% Tween 20) at 37°C for 1 h. CovR was detected by the anti-CovR serum (Chiang-Ni et al., 2016), and SLO was detected by the anti-SLO antibody (GeneTex; Irvine, CA, United States). The phosphorylated and nonphosphorylated CovR and SLO were visualized using a previously described method (Chiang-Ni et al., 2020).

### Whole Blood Model

The growth of GAS strains in human whole blood was studied according to a previous study with modifications (Brouwer et al., 2020). Freshly drawn heparinized venous blood from healthy adults was aliquoted (360 μl) into wells of a 24-well plate. GAS strains were grown to the exponential phase (OD_600_ = 0.6) in TSBY, washed, resuspended in 1× PBS buffer at ~5 × 10^5^ colony forming unit (CFU)/ml, and added to whole blood in a final volume of 400 μl (~5 × 10^4^ CFU/ml). After incubating for 1.5 h at 37°C, the growth of GAS strains was analyzed by plating serial dilutions on TSBY plates. Experiments were performed with blood from three different donors.

### Phage Induction, Infection, and Lysogenic Conversion

Mitomycin C (0.2 μg/ml; Sigma-Aldrich; St. Louis, MO, United States) was added to the culture of donor strain grown to the early exponential phase and incubated at 37°C for another 4 h. Culture supernatants were passed through a 0.45 μm filter (Millipore Ireland Ltd., Co.; Cork, Ireland) to remove bacteria and large fragments, and phage particles were collected from the filtrate by ultra-centrifugation at 112,000 × *g* for 2 h at 10°C. The recipients were grown to the logarithmic phase in the presence of 5 mM CaCl_2_ and then co-incubated with the phage particles at 37°C for 3 h. The bacterial suspension was plated on agar plates supplemented with chloramphenicol (3 μg/ml) and resistant convertants were collected for Southern blotting analysis.

### Statistical Analysis

Statistical analysis was performed using Prism software, version 5 (GraphPad; San Diego, CA, United States). Significant differences between multiple groups were determined using the ANOVA. Post-test for ANOVA was analyzed using Tukey’s honestly significant difference test. Statistical significance was set at *p* < 0.05.

## Results

### Prevalence of *emm* Type, *ssa*+, and Erythromycin-Resistant GAS Isolates in 2008–2019

**Figure 1:**
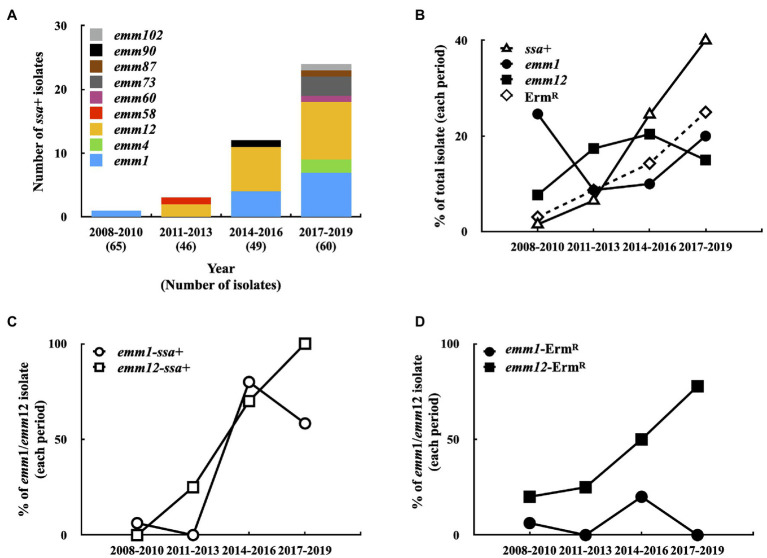
Prevalence of *emm* type, *ssa*+, and erythromycin-resistant (Erm^R^) isolates in 2008–2019. **(A)** The *emm* type of *ssa*+ isolates in 2008–2019. **(B)** Prevalence of *emm*1-type, *emm*12-type, *ssa*+, and Erm^R^ isolates in 2008–2019. **(C)** Prevalence of *emm*1 and *emm*12 *ssa*+ isolates in 2008–2019. **(D)** Prevalence of *emm*1 and *emm*12 Erm^R^ isolates in 2008–2019.

A total of 34 different *emm* types were identified among 220 isolates, among which *emm*1 (37/220; 16.8%), *emm*12 (32/220; 14.5%), *emm*113 (20/220; 9.1%), *emm*102 (16/220; 7.3%), *emm*11 (14/220; 6.4%), and *emm*90 (14/220; 6.4%) accounted for 60.5% total isolates. Phage-encoded *ssa* was detected by PCR. One isolate was *ssa*+ in 2008–2010 (1/65, Figure 1A); however, *ssa*+ isolate prevalence increased to 6.5% (3/46) in 2011–2013, 24.5% (12/49) in 2014–2016, and 40% (24/60) in 2017–2019 (Figure 1B). The most prevalent *emm* types of *ssa*+ isolates were *emm*12 (18/40; 45%) and *emm*1 (12/40; 30%). Although *emm*1 and *emm*12 isolate prevalence did not dramatically increase during 2008–2019 (Figure 1B), that of *ssa*+ *emm*1 isolates increased to 58.3% (7/12) in 2017–2019 compared to 6.3% (1/16) in 2008–2010 and that of *ssa*+ *emm*12 isolates increased to 100% (9/9) in 2017–2019 compared to 0% (0/5) in 2008–2010 (Figure 1C).

Among the 220 isolates, 28 (12.7%) were erythromycin-resistant. The prevalence of erythromycin-resistant isolates gradually increased from 3.1% (2/65) in 2008–2010 to 8.7% (4/46) in 2011–2013, 14.3% (7/49) in 2014–2016, and 25% (15/60) in 2017–2019 (Figure 1B). PCR analysis showed that *mefA* was detected in one erythromycin-resistant isolate (*emm*12 type), and the remaining 27 erythromycin-resistant isolates were either *ermB*+ (22 isolates) or *ermTR+* (5 isolates), indicating that these isolates were also clindamycin-resistant (Supplementary Table S1). In addition, 53.6% (15/28) of erythromycin-resistant isolates were type *emm*12; the prevalence of erythromycin-resistant *emm*12 isolates increased from 20% (1/5) in 2008–2010 to 77.8% (7/9) in 2017–2019 (Figure 1D). Moreover, 77.8% (14/18) *ssa*+ *emm*12 isolates were erythromycin-resistant and 92.9% (13/14) *ssa*− *emm*12 isolates were erythromycin-susceptible. Although the prevalence of the *ssa*+ *emm*1 isolates was increased (Figure 1C), only two isolates (total 37 isolates) were erythromycin-resistant. These results indicate that the prevalence of *ssa*+ isolates, particularly *emm*1 and *emm*12 isolates, increased. Most of the erythromycin-resistant *emm*12 isolates were *ssa*+ (14/15); however, 94.6% (35/37) *ssa*+ *emm*1 isolates were erythromycin-susceptible.

### CovR/CovS Negatively Regulates *ssa* Transcription

**Figure 2:**
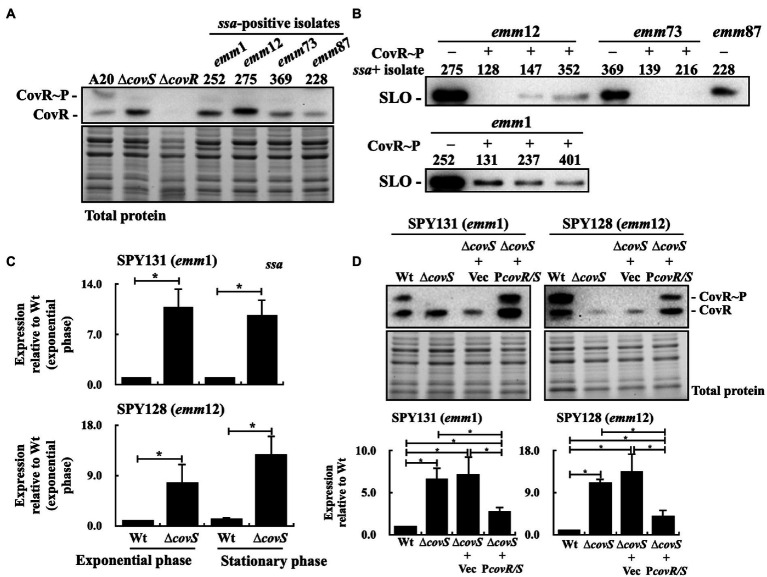
Phosphorylated CovR and streptolysin O (SLO) expression and *ssa* transcription in selected clinical isolates, *covS* isogenic mutants, vector-control strains, and *covR/covS trans*-complementary strains. **(A)** Detection of the phosphorylated CovR in the selected clinical isolates using Phostag western blot assay. A20 and its *covS* (∆*covS*) and *covR* (∆*covR*) mutants were utilized as experimental controls. **(B)** SLO secretion in selected *ssa*-positive isolates. SLO was detected in the culture supernatants using western blot analysis. **(C)** *ssa* transcription in the SPY131 (*emm*1) and SPY128 (*emm*12; Wt) and their *covS* mutants (∆*covS*). **(D)** Phosphorylated CovR expression and *ssa* transcription in vector-control (∆*covS*+Vec) and the *covR/covS trans*-complementary (∆*covS*+P*covR/S*) strains. RNA was extracted for reverse transcription-PCR (RT-qPCR) analysis. ^*^p < 0.05. CovR~P, phosphorylated CovR; CovR, nonphosphorylated CovR. Total protein is served as the internal loading control.

Brouwer et al. (2020) showed that SSA is a thiol-activated superantigen, and its release and activity are promoted by the pore-forming toxin SLO. Moreover, the isolates from patients with invasive infection obtained spontaneous mutations in the *covR/covS* operon more frequently than those from patients with pharyngeal/tonsil infection (Ikebe et al., 2010; Friaes et al., 2015). Spontaneous inactivating mutations in *covR*/*covS* cause loss of CovR phosphorylation resulting in derepression of multiple secreted virulence factors including SLO (Sumby et al., 2006; Chiang-Ni et al., 2019b). In this study, after analyzing CovR phosphorylation level in 220 isolates using Phostag western blot assay, phosphorylated CovR was absent in 29 isolates (data not shown; Supplementary Table S1). Among the 40 *ssa*+ isolates, we identified four isolates that did not express the phosphorylated CovR protein (Figure 2A). Also, these four isolates expressed higher levels of SLO than those with the phosphorylated CovR protein (Figure 2B).

To investigate the effect of CovR phosphorylation inactivation on the *ssa* expression, isogenic *covS* mutants of *emm*1-type (SPY131) and *emm*12-type (SPY128) isolates were constructed, and *ssa* transcription was analyzed by quantitative PCR. The results showed that *ssa* expression in the *covS* mutants was 7–12-fold higher than that in the parental strains (Figure 2C). To further verify that *ssa* expression is repressed by phosphorylated CovR, the *covR/covS trans*-complementary strains of the *covS* mutants were constructed, and the phosphorylated CovR and *ssa* transcription in the *covR/covS trans*-complementary strains were analyzed. Phostag western blot analysis showed that phosphorylated CovR was detected in the *trans*-complementary strains, but not in the vector-control strains and *covS* mutants (Figure 2D). In addition, *ssa* transcription was repressed in the *covR/covS trans*-complementary strains compared to that in the *covS* mutants (Figure 2D). These results indicate that *ssa* transcription is repressed by phosphorylated CovR.

### The Role of SSA on *covS* Mutant Survival in Human Whole Blood

**Figure 3:**
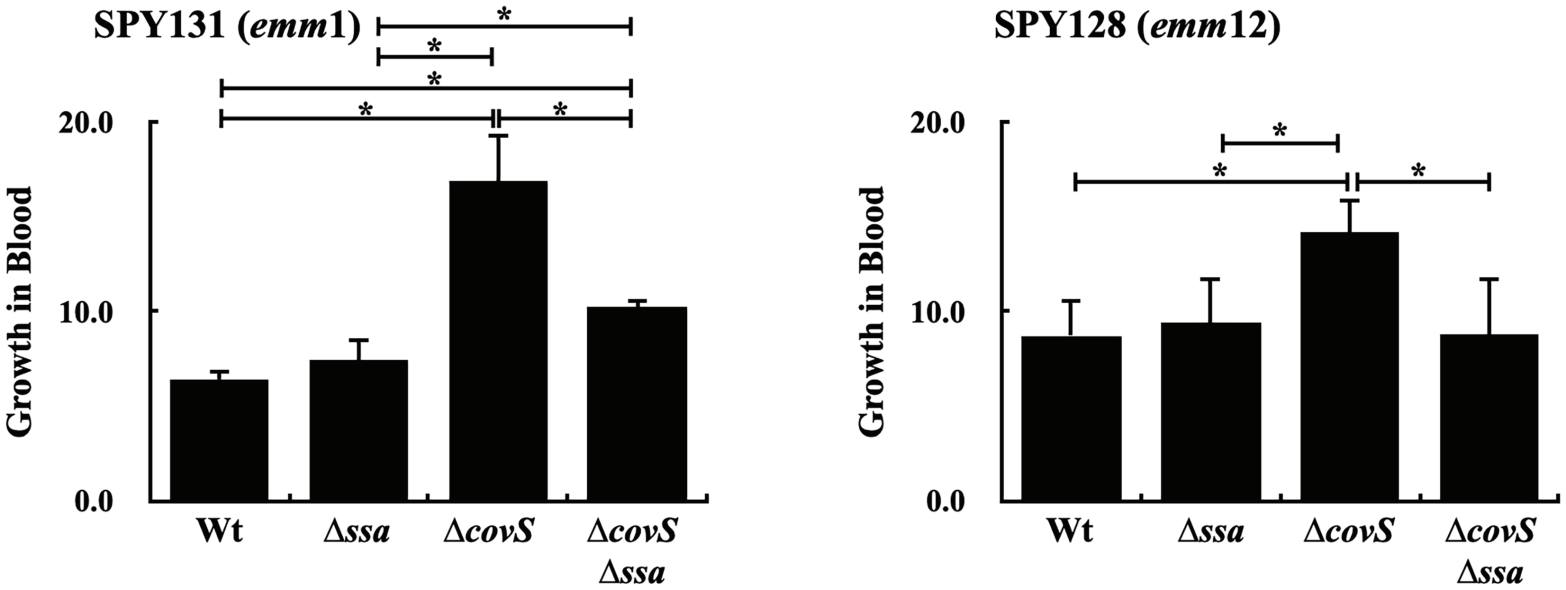
Growth activity of SPY131 (*emm*1), SPY128 (*emm*12), their *ssa* mutants (∆*ssa*), *covS* mutants (∆*covS*), and *covS* and *ssa* double mutants (∆*covS*∆*ssa*) in human whole blood. Group A *Streptococcus* (GAS) strains were incubated with whole blood from donors for 1.5 h. The number of surviving bacteria in human blood was determined by plating and enumerating the colony forming units (CFUs), which determined growth relative to the initial inoculum. ^*^p < 0.05.

Brouwer et al. (2020) showed that the wild-type strain and *ssa* mutant had similar growth in human whole blood. *ssa* transcription and SLO production were upregulated in the *covS* mutants compared to the wild-type strains (Figures 2B,C); therefore, in this study, the role of SSA in *covS* mutant survival in human whole blood was further investigated. The *ssa* and *covS* double mutants of *emm*1-type SPY131 and *emm*12-type SPY128 were constructed. The growth of the wild-type strains, *covS*, and *ssa* mutants in the culture broth were similar (data not shown). *covS* mutants were more resistant to phagocytic killing (Sumby et al., 2006); in agreement with this, we observed that the *covS* mutants of SPY131 and SPY128 had better growth in human blood than the wild-type strains and *ssa* mutants (Figure 3). Furthermore, the results showed that *ssa* deletion in the *covS* mutants significantly attenuated bacterial growth in blood compared to the *covS* mutants (Figure 3), suggesting that SSA contributes to the survival of *covS* mutants in human whole blood.

### Transfer of *ssa* by Phage

**Figure 4:**
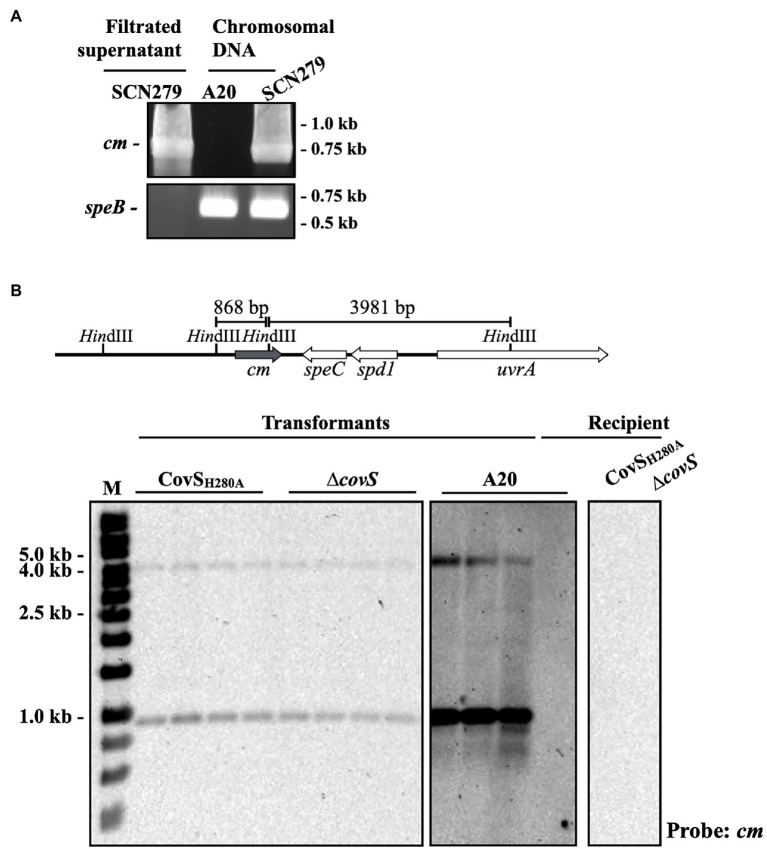
PCR and Southern blot analyses of the *ssa* mutant and lysogenic convertants. **(A)** Detection of the chloramphenicol cassette (*cm*) and the chromosomal *speB* gene in the chromosomal DNA and filtrated supernatant after mitomycin C treatment using PCR. SCN279, the phage donor. A20 (*emm*1-type), the recipient. **(B)** Southern blot hybridization of lysogenic convertants. The upper panel shows the *Hin*dIII site and the predicted sizes for *cm* probe hybridization according to ΦHKU.vir (HKU360, NCBI accession no. CP009612) and A20 (NCBI accession no. CP003901.1) sequence. The recipient strains (A20, CovS_H280A_, and ∆*covS*) showed no signal for *cm* probe hybridization and used as the experimental negative control. M, DNA marker.

The *ssa*+ isolates that did not express the phosphorylated CovR protein were identified (Figure 2A). These isolates could acquire inactivating spontaneous mutations in *covR/covS* during infection. Nonetheless, the *ssa* gene is carried by phage and the possibility of *covR*/*covS* mutants directly acquired *ssa* by phage infection could not be excluded. Therefore, whether *covS* mutants could acquire *ssa* through phage infection and lysogenic conversion was further investigated. To select the lysogenic convertants after phage infection, *ssa* was replaced with the chloramphenicol (*cm*) cassette in the *emm*12-type SPY128 (SCN279), which was utilized as the *ssa-*encoding phage donor. After mitomycin C induction, the *cm* cassette, but not chromosomal *speB*, was detected in the filtered supernatant from SCN279 (Figure 4A), indicating that the target phage was released from SCN279. Next, the recipients, including the *emm*1-type A20 strain, its *covS* mutant AP3, and the CovS kinase-inactivated CovS_H280A_ mutant (Chiang-Ni et al., 2016, 2019b), were incubated with the collected phage particles. We found that lysogenic convertants can be obtained. The *cm* cassette in the lysogenic convertants was integrated to the same insertion site of the chromosome and located on the same phage in donor strain SCN279 (Figure 4B). These results indicate that not only *emm*1 wild-type strain but also its *covS* mutants could acquire *ssa* by lysogenic conversion.

## Discussion

This study showed that the prevalence of *ssa*+ GAS isolates increased from 1.5% (1/65) in 2008–2010 to 40% (24/60) in 2017–2019 in northern Taiwan. A total of 29 isolates could not produce phosphorylated CovR protein, and four of these isolates were *ssa*+. We found that *ssa* transcription is repressed by phosphorylated CovR. Moreover, *ssa* gene deletion attenuated the growth activity of the *covS* mutants in human blood, suggesting that the acquisition of *ssa* by *covR/covS* mutants or spontaneous mutations in the *covR/covS* operon in the *ssa*+ isolates could be related to the increase in bacterial survival during infection.

In this study, 10% *ssa*+ isolates did not produce phosphorylated CovR under the conditions tested. Spontaneous inactivating mutations in the *covR/covS* operon increases SLO expression in GAS (Sumby et al., 2006; Chiang-Ni et al., 2019a). In addition, some clinical isolates with spontaneous mutations or a truncated allele of *rocA* (regulator of cov; upstream regulator of CovR/CovS) also increased the SLO expression (Feng et al., 2017; Jain et al., 2017; Horstmann et al., 2018; Chiang-Ni et al., 2020). SSA is a thio-activated superantigen, and the GAS secreted SLO, which triggers glutathione release from host cells to activate SSA *in vivo* (Brouwer et al., 2020). Brouwer et al. (2020) showed that *ssa* deletion did not attenuate bacterial survival in human blood; however, our results showed that the *covS* and *ssa* double mutants had significantly decreased growth in human blood compared to the *covS* mutants (Figure 3). As expected (Sumby et al., 2006), the encapsulated *covS* mutant was resistant to phagocytosis (Figure 3), thus not cleared despite high expression (promoted by SLO) of the superantigen SSA, which might activate T cells and phagocytic cells. Okamoto et al. (2001) showed that macrophages in SEB-pretreated mice were less phagocytic than those in non-pretreated mice. SSA sequence is 60% identity to SEB. Based on these observations, although *covS* mutant and *covS* and *ssa* double mutant were both encapsulated, the *covS* and *ssa* double mutant might encounter higher pressure from macrophages than that of *covS* mutant. Nonetheless, these mutants were co-cultured with human whole blood for 1.5 h; whether SSA could act to macrophages like SEB during this short incubation period is not clear. Therefore, the role of SSA in GAS survival in human blood needs to be further elucidated. Whether the *ssa* transcription is directly regulated by phosphorylated CovR is not clear; however, our results suggest that SSA could potentially contribute to the pathogenesis of invasive *covR/covS* mutants.

Lynskey et al. (2019) showed that the toxigenic M1T1 clone (M1T1_UK_) is related to the increased incidence of invasive GAS disease in the United Kingdom. Thereafter, the M1T1_UK_ strain was identified in Canada, the Netherlands, and the United States (Demczuk et al., 2019; Li et al., 2020b; Rumke et al., 2020), demonstrating the possibility of clonal expansion of the M1T1_UK_ clone. In Taiwan, Yan et al. (2000) showed that 64.3% erythromycin-resistant isolates were clindamycin-susceptible and harbored *mefA* before 1998. From 2001 to 2010, the erythromycin resistance rate decreased from 53.1 to 0% in southern Taiwan (Chuang et al., 2015). In this study, we found that the erythromycin resistance rate in GAS increased from 3.1% in 2008–2010 to 25% in 2017–2019. Notably, only one isolate harbored *mefA* (1/28). The changes in phenotype and genotype of erythromycin-resistant GAS suggested that the expansion of erythromycin-resistant (*ermB* and *ermTR*) and *ssa+ emm*12 clones could occur in Taiwan. In the phage infection assay, *ssa* was replaced with a chloramphenicol cassette as the selection marker, and an identical genetic element was found in the *emm*12-type donor and *emm*1-type recipient strains using Southern blot (Figure 4B and data not shown), suggesting that *ssa* could be transferred by phage. Furthermore, we also found that the chloramphenicol cassette could be transferred between *emm*12-type and *emm*73-type isolates (data not shown). These results suggest that clonal expansion and phage infection could be involved in the increased prevalence of *ssa*+ isolates in Taiwan. The emerged clones in the United States (M1T1 clone) and in Asia (Hong Kong and Taiwan, *emm*12 clone) are different, but both possess superantigen genes such as *speA* and *ssa*. Nonetheless, the exact role of superantigens in the survival fitness of GAS during infection needs to be addressed.

In summary, this study showed an increased prevalence of *ssa*+ and erythromycin-resistant GAS isolates in northern Taiwan, and demonstrated that *ssa* acquisition by invasive *covS* mutants or spontaneous mutations in the *covR/covS* operon of *ssa*-positive isolates could enhance bacterial growth in human blood. The role of SSA in GAS pathogenesis, particularly in invasive *covR/covS* mutants, should be further investigated.

## Data Availability Statement

The original contributions presented in the study are included in the article/**Supplementary Material**, further inquiries can be directed to the corresponding author.

## Ethics Statement

The studies involving human participants were reviewed and approved by the Institutional Review Board (201900274B0 and 202000479B0) of the Chang Gung Memorial Hospital, Taiwan. The patients/participants provided their written informed consent to participate in this study.

## Author Contributions

CC-N, Y-SL, and C-YL contributed to the conceptualization, methodology, formal analysis, and writing the original draft. Y-SL, C-YL, C-YH, and Y-AS contributed to the methodology and investigation. Y-YC and C-HL contributed to the conceptualization and methodology. CC-N contributed to the writing – review and editing. CC-N and C-HC contributed to the funding acquisition. All authors contributed to the manuscript revision, read, and approved the submitted version.

### Conflict of Interest

The authors declare that the research was conducted in the absence of any commercial or financial relationships that could be construed as a potential conflict of interest.

## Footnotes

^1^https://www.cdc.gov/streplab/groupa-strep/emm-typing-protocol.html
